# A National Study of Nutrition and Nutritional Status of the Adult Polish Population in the Years 2017–2020 before and during the COVID-19 Pandemic—Design and Methods

**DOI:** 10.3390/nu13082568

**Published:** 2021-07-27

**Authors:** Iwona Traczyk, Filip Raciborski, Alicja Kucharska, Beata I. Sińska, Magdalena Milewska, Bolesław Samoliński, Dorota Szostak-Węgierek

**Affiliations:** 1 Department of Human Nutrition, Faculty of Health Sciences, Medical University of Warsaw, 27 Erazma Ciołka Street, 01-445 Warsaw, Poland; iwona.traczyk@wum.edu.pl (I.T.); beata.sinska@wum.edu.pl (B.I.S.); 2 Department of Prevention of Environmental Hazards and Allergology, Faculty of Health Sciences, Medical University of Warsaw, 1a Banacha Street, 02-091 Warsaw, Poland; filip.raciborski@wum.edu.pl (F.R.); boleslaw.samolinski@wum.edu.pl (B.S.); 3 Department of Clinical Dietetics, Faculty of Health Sciences, Medical University of Warsaw, 27 Erazma Ciołka Street, 01-445 Warsaw, Poland; mmilewska@wum.edu.pl (M.M.); dorota.szostak-wegierek@wum.edu.pl (D.S.-W.)

**Keywords:** Polish population, nutrition, obesity, physical activity, health, sarcopenia, COVID-19

## Abstract

The paper presents the designs and methods of a cross-sectional study of two groups of randomly selected Polish inhabitants aged 19–64, and 65 and over, carried out as part of the National Health Program. The aim of the study was to illustrate the current health situation of the respondents in terms of nutrition and physical activity level. The quantitative and qualitative methods were used. The Computer Assisted Personal Interview technique was used. The dietary research was carried out through repeated interviews about the frequency of food consumption, and about what food had been consumed in the previous 24 h. In addition to the questionnaire studies, anthropometric data, blood pressure and the level of physical activity were measured. During the COVID-19 pandemic, some methods were modified according to hygiene rules. The Computer Assisted Telephone Interview technique was used to collect the data, and the anthropometric data were obtained via measurements made by the respondents themselves based on detailed instructions. The results will be used to present representative data for the Polish population, describing a wide range of eating behaviours and other lifestyle elements, food and nutrition knowledge, dietary supplement use, the occurrence of diet-related diseases, nutritional status and, in the seniors group, the risk of sarcopenia.

## 1. Introduction

The greatest negative impact on the health and well-being of the population in the WHO’s European Region is due to nutritional factors, mainly excessive consumption of saturated fat, trans-unsaturated fatty acids, sugar and salt. Another problem is that too few vegetables, fruit, fish and whole grain cereals are consumed. Over the past several decades, many countries in the WHO’s European Region have recorded an increase in rates of overweight and obesity [1].

According to data from the World Health Organization (WHO), in 2016 excess body weight was present in over 1.9 billion of the adult world population, including over 650 million with obesity. Among the reasons for this situation, WHO lists increased consumption of food with high energy density, high fat and sugar content, as well as insufficient physical activity [1]. The major risk factors for cardiovascular disease worldwide are elevated blood pressure, which is responsible for 19% of global deaths annually; excess body weight, hypercholesterolemia, smoking, and elevated blood glucose levels. Annually, over 1.6 million people worldwide die due to insufficient physical activity [2].

These phenomena also apply to Poland. In the Second Multicenter National Population Health Survey (WOBASZ II), carried out in 2013–2014, excessive body weight was found in 77.6% of men and 55.5% of women, including obesity in 24.4% of men and 30.5% of women [3]. A lower percentage of the Polish population with excess body weight was shown in the European Health Survey (EHIS from 2014), in which too much body weight was recorded in 62% of adult men and 46% of women, including obesity in 15.6% of women and 18.1% of men [4]. However, attention should be paid to the differences in the methodology of both studies. In the WOBASZ II study, the respondents were weighed by nurses, while the EHIS study relied on the self-reports of the subjects.

Next, similarly to the WOBASZ II study, it was stated in the report from The EU-SILC survey of 2017 (published in 2018 by the Central Statistical Office (GUS)), that excessive body weight occurred in 77.3% of the Polish population in aged 16 and more, including obesity in 32.4% of respondents [5]. The 2019 research results published by CBOS (Public Opinion Research Centre), meanwhile, indicate a lower percentage of people with excess body weight (59%); obesity was diagnosed in one in five Poles [6]. It is worth noting here that these data were also based on the self-reports of the respondents.

In addition to improper nutrition, insufficient physical activity is an important risk factor for the development of overweight and obesity and other chronic diseases. A low level of physical activity may result in an increase in mortality from cardiovascular diseases and malignant cancers. This risk factor is also very common in Poland. The inhabitants of Poland remain one of the least physically active populations in the European Union, although some small improvement has been observed in recent years. While the research from 2015 indicated that 6 out of 10 Poles did not engage in any physical activity [7], the results of studies published in 2018 [8] showed that 61% of the respondents practiced sports. These results were confirmed by the MultiSport Index study of 2019 [9], in which 64% of the surveyed Poles reported practicing physical activity. These research results concerning Poland above are based on surveys of selected groups of Polish residents or relate to the status of several years ago.

This work presents the scope of research and the main methods used in cross-sectional studies on the diet and nutritional status of two Polish populations: adults aged 18–64, and seniors—people aged 65 and over. Two thousand respondents were included in the study in each age group. The research was conducted in line with the public health strategy being developed in Poland, which is reflected in the executive act to the Public Health Act [10], i.e., the Regulation of the Council of Ministers on the National Health Program (NHP) for 2016–2020 [11]. The strategic goal of the NHP is to extend healthy life, improve the health and related quality of life of the population, and reduce social inequalities in health. The improvement of diet, nutritional status and physical activity of the society were among the 6 main goals of the Program.

The research was carried out from 2017 to 2020 at the Faculty of Health Sciences of the Medical University of Warsaw (MUW) at the request of the Ministry of Health.

The purpose of the methods and measurements used was to provide current data on the occurrence of selected risk factors for obesity and other diet-related diseases among the adult residents of Poland. The factors selected were: improper diet, low physical activity, excessive body weight, hypertension, food and nutrition beliefs, and for the group of seniors, the rate of sarcopenia risk.

This work, in addition to the description of the methods used, includes an announcement of new research results which are valid for a representative Polish population. The results are used to present uniquely representative data for the Polish population, describing a wide range of eating behaviours and other lifestyle elements, knowledge about food and nutrition, taking dietary supplements, the occurrence of diet-related diseases, including obesity, and the risk of sarcopenia among seniors.

## 2. Materials and Methods

### 2.1. Sample Characteristics and Selection

The study targeted the adult population residing on the territory of the Republic of Poland. The study population was divided into the following two groups: adults aged 19–64 years (NHP-A (adults)) and senior citizens aged 65 years and over (NHP-S (seniors)).

The study was approved by the Institutional Ethical Review Board at the Medical University of Warsaw (Approval Nos AKBE/163/17 and AKBE/164/17). The execution of the study complied with the provisions of the General Data Protection Regulation [12].

When selecting respondents, the guidelines of the European Food Safety Authority (EFSA) [13] were followed. The selection was made on the basis of the address database of residential premises in Poland. The sampling was probabilistic (random). Additional techniques were employed to enhance the representativeness of the sample (stratified sampling) and to reduce the cost of the field study (cluster sampling).

A two-stage sampling scheme was used. The first stage involved randomly selecting 500 (out of a total of 34,633) statistical areas, i.e., territorial units established by Polish Central Statistical Office for research purposes. Each statistical area represented a separate cluster (cluster sampling). Stratified sampling was also used in the process, with strata based on information about a given voivodeship (major administrative region) and type of gmina (minor administrative division). The probability of a particular cluster being selected for the study was proportional to the number of residents.

In the second stage, residential buildings were selected within a cluster. Buildings were identified with the TERYT-NOBC register (TERYT—the National Official Register of the Territorial Division of the Country—led by the Polish Central Statistical Office in electronic form, NOBC is a part of TERYT and is a system for address identification of streets, real estate, buildings and dwellings) and other external data sources [14]. Information about the number of separate apartments and number of individuals officially residing there, stratified by age group, was also used. Eight buildings were selected within each cluster (4 for the NHP-A group and 4 for the NHP-S group) together with additional buildings that comprised a reserve sample in case a given respondent refused to participate or an interview could not take place. The actual respondents were individuals selected from each building previously drawn (one respondent per building). 

In March 2020, in the wake of the COVID-19 pandemic, the study methodology had to change (while the study was on-going) from computer assisted personal interviews (CAPI) to computer assisted telephone interviews (CATI).

The original sample did not involve data on the potential respondents’ telephone numbers. For the purposes of telephone interviews, a two-stage sampling selection scheme with stratified sampling was prepared. Strata were defined with regard to the division into conventional vs mobile phones. In the NHP-S group, the stratum of conventional phones was overrepresented compared to the proportion of conventional telephones in the population at large. For users of conventional telephones, the approximate addresses of potential respondents are known. Sample selection in the group of users of conventional telephones included an additional division into strata (voivodeships). As one conventional telephone may be used by more than one individual, respondents were selected from among the users of a given telephone number based on the inclusion criteria (age). If several individuals met the age criterion, the person ultimately selected was the one who had celebrated their birthday the most recently.

For the stratum of mobile telephones, simple random sampling was performed on the entire pool of available telephone numbers. The user’s place of residence and age were determined during a preliminary conversation. If the respondent met the inclusion criteria, the interview proper followed.

The selection of a sample of telephone numbers utilised a database of all telephone numbers (mobile and conventional) active in Poland (served by telephone operators registered in Poland) generated using the Office of Electronic Communications’ number tables. Diagrams illustrating the sampling methodologies are given in a supplementary file, Figures S1 and S2.

### 2.2. Methods

Both quantitative and qualitative methods were employed.

A number of questionnaire-based techniques and anthropometric measurements were used, together with measurements of blood pressure and physical activity levels, the latter with Actigraph GT3X triaxial accelerometers. Blood pressure measurement and all applied anthropometric methods were validated or conducted in accordance with WHO guidelines. Physical activity levels were measured by questionnaires and accelerometers. With regard to the part of the survey concerned with nutrition, the methodology was substantially based on EFSA’s guidelines published in connection with the EU Menu project [13,15] and on the recommendations of the Committee of Human Nutrition of the Polish Academy of Sciences [16,17]. The research tool included some questions from the Dietary Habits and Nutrition Beliefs Questionnaire (KomPAN) [18], used in nutrition research in Poland, to investigate the frequency of food consumption, eating habits, and respondents’ knowledge. The KomPAN questionnaire was developed by experts from the Committee of Human Nutrition, Polish Academy of Sciences.

A number of other questionnaires and measurements were also used. Altogether, the research tool consisted of 11 identical components for both groups (NHP-A and NHP-S), with senior citizens being additionally asked questions from a screening questionnaire developed to assess the risk of sarcopenia (Simple Questionnaire to Rapidly Diagnose Sarcopenia, SARC-F) [19].

Table 1 contains information about the techniques and measurements used and the order in which they were administered. Data were collected at respondents’ homes during 2 visits over 5–7 days, with the second visit involving only the diet-related section (24-h dietary recall interview) and a blood pressure measurement. The field study was conducted between October 2017 and June 2020. Before the COVID-19 pandemic, all survey components were supported with CAPI, which was replaced with CATI following the onset of the pandemic. Electronic data collection forms were also used to capture all results of anthropometric and blood pressure measurements.

#### 2.2.1. 24-h Dietary Recall Interview

The 24-h dietary recall interview was our core research tool. 24-h dietary recall is a widely used qualitative-quantitative method that provides information about the composition of meals and the amount of food products or nutrients consumed. 

In keeping with EFSA recommendations [13], the interview was administered to each respondent twice over a span of at least 5 days. In order to account for the variability of eating habits within the week, interviews were administered on different days of the week. The interviews used the CAPI technique supported by DIETA 5.0, an application developed at National Food and Nutrition Institute in Warsaw and used for research purposes in Poland [24]. DIETA 5.0 uses data on the nutritive value of food products and dishes and national nutrition standards. Serving sizes for food products and dishes were described quantitatively using household measures (e.g., glasses, cups, spoonfuls, etc.), food servings (retrieved from manufacturers’ information) and an album of serving sizes of food products and dishes [23]. The application is used for calculating energy and nutritive value of particular diets and comparing the diets to nutrition standards; it ensures standardization with respect to: the technique of entering data on food products and dishes consumed (sequence and precision in specifying the type and composition of a dish); recipes for dishes; description of serving sizes (a list of serving sizes is suggested by the application); calculation technique (identical reduction rates) and results (number of ingredients, comparison against standards). 

The programme DIETA allows an unlimited number of interviews or food consumption records to be entered for an individual. As a new version (DIETA 6.0) of the application was launched while the study was ongoing, all previously entered data were converted to the DIETA 6.0 format and recalculated. DIETA 6.0 accounts for more than 2100 dishes and food products, including seafood, enriched products, gluten-free products, mineral waters, fast food products, nutritive baby foods, energy and isotonic drinks and more than 1290 dietary supplements [23,24]. 

The DIETA 5.0/6.0 programme converted the data on food products consumed to energy and nutrient content data with its internal databases.

#### 2.2.2. Frequency of Food Consumption

In order to identify the individual variability in food consumption (e.g., holidays) and minimize its impact on the results obtained, the food frequency questionnaire (FFQ) method was used. A simplified food frequency questionnaire (FFQ) based on the KomPAN questionnaire was used [18]. The questionnaire comprised 24 questions on how frequently healthful food (such as vegetables, fruit, wholemeal cereal products, dairy products and fish), and products whose consumption should be limited (such as red meat, refined flour products, fast foods, sweets, alcoholic beverages), were consumed. Respondents chose one out of 6 possible responses: never, 1–3 times a month, once a week, several times a week, once a day, several times a day.

#### 2.2.3. Measurement of Physical Activity Level

Physical activity level was measured with two independent tools: a device for monitoring physical activity and a research questionnaire. 

#### 2.2.4. Measurement with a Physical Activity Monitor

A physical activity monitor was used to measure actual levels of physical activity of the respondents. The Actigraph triaxial accelerometer model Gt3x (Actigraph Corp., Pensacola, FL, USA) was used to assess physical activity, with an epoch length of 60 s and a frequency rate of 30 Hz. The measurements were collected between the two study visits at respondents’ homes. During the first visit, interviewers instructed study participants how to use the devices and regular initialization primed the monitors to prepare them for registering data. Participants were asked to wear a monitor over the hip for 5–7 days (24-h protocol), removing it only for water-based activities. Non-wear time was filtered from the raw data using Choi’s 2011 [27] definition and excluded from further analysis. Ninety minute bouts or more of consecutive zero counts were classified as non-wear time; during these periods, intervals of up to 2 min of non-zero counts were also classified as non-wear time. Data were included in the analysis if participants had at least 10 hours of wear time daily.

Data were analysed centrally to ensure standardization. Raw data were integrated into 60 s epochs and used to derive counts per minute (CPM). Energy expenditure was calculated based on Freedson Combination 98 algorithm, where Work-Energy Theorem is used to calculate the caloric expenditure below 1951 counts and an algorithm developed by Freedson in 1997 is used to calculate the energy expenditure above 1952 counts [27]. The MET rate was calculated with the Freedson Adult equation [28]. The time involved in bouted moderate-to-vigorous physical activity (MVPA) (periods of at least 10 continuous min of MVPA) was calculated based on a PA recommended vector magnitude cut point of ≥1952 counts/min (up to 2 min below the cut point allowance), and it was expressed in min/day [28]. Sedentary time was calculated as the amount of time accumulated below 100 counts/min and was expressed in min/day. Additionally, raw data were converted to counts per minute using an LFE (low frequency extension) filter to explain the differences between the two ways of filtering data (with and without LFE filter) [29]. The data download, reduction, cleaning and analyses were performed using ActiGraph software (ActiLife Version. 6.11.9; Actigraph).

#### 2.2.5. Physical Activity Interview

Physical activity was additionally assessed with a survey. The main questionnaire was the abridged IPAQ questionnaire (International Physical Activity Questionnaire (IPAQ-SF), recommended by the European Commission for use in national and regional surveys [25]. Two questions from the KomPAN were additionally used [18]. 

After a 3-month hiatus (March–May 2020), questionnaire-based acquisition of physical activity data was resumed by telephone (CATI).

#### 2.2.6. Blood Pressure Measurements

Blood pressure measurements were performed during both study visits to respondents’ homes. Interviewers used M2 Basic Omron sphygmomanometers. Blood pressure was measured with the respondent sitting down after at least 5 min of rest. The cuff was placed on the respondent’s right arm 2–3 cm above the elbow flexure. In the presence of medical contraindications (e.g., a history of mastectomy or another surgical procedure), the left arm was used. Mean values from the two measurements were used in subsequent statistical analyses.

The blood pressure data served to detect hypertension in the study population based on the classification presented by European Society of Cardiology and the European Society of Hypertension [21].

#### 2.2.7. Anthropometric Measurements

The respondents’ height and body weight and waist and hip circumferences were measured in line with WHO recommendations [30]. These measurements were carried out during the first study visit. The measurements were performed according to the scheme shown in Table 2, accounting for differences in health situations and physiological status of the patients.

In order to make the circumstances more comfortable for the respondents, before the COVID-19 pandemic, measurements for female respondents were taken by female interviewers, and by male interviewers for male respondents.

#### 2.2.8. Body Weight and Height

Body weight was measured with portable electronic scales from Omron (model HBF-212). Respondents were weighed in light clothes with empty pockets and no shoes on, to an accuracy of 0.1 kg.

Height was measured with a graduated anthropometric tape for linear measurements and a set square. A stadiometer was not used because the measurements were obtained in the field study setting. For the measurement, respondents were wearing no shoes and were standing upright with the back to the wall, feet joined together and head in the Frankfurt plane position. The interviewer placed a sticky note on the wall above the tip of the respondent’s nose so that the respondent would touch the piece of paper with the back of their head. Then, the interviewer placed the set square at the top of the respondent’s head at a right angle to the wall and marked a point or drew a line with a pencil at the level of the lowest-lying edge of the set square. The distance between the point or line so marked and the floor was measured with the measuring tape to an accuracy of 0.1 cm.

#### 2.2.9. Waist and Hip Circumferences

If the respondent agreed to the procedure, these measurements were performed directly on the skin (with clothing removed). If the respondent was wearing clothing during the measurement, this was noted down by the interviewer.

For the measurement of waist circumference, the respondent stood up straight with the abdominal muscles as relaxed as possible. The interviewer positioned the tape around the respondent’s abdomen at the narrowest point, parallel to the floor, making sure the tape was not twisted or stretched too tight. The results were noted to an accuracy of 0.1 cm. If no definite narrowing could be seen at the waist, the measurement was performed 3 cm above the navel.

The measurement of hip circumference differed slightly between men and women on account of anatomical differences. In women, the broadest part of the hip is found just above the pubic bone, while in men, it is seen between the iliac crest and pubic bone. Accordingly, when performing the measurement in a woman, the interviewer placed the measuring tape around the hip at the level of the widest gluteal circumference and in men, the landmark was the upper end of the femur. The result was recorded to an accuracy of 0.1 cm. 

#### 2.2.10. Anthropometric Indices of Nutritional Status

The results of anthropometric measurements were used to calculate indices for assessing the nutritional status and the presence of abdominal obesity. For this purpose, the BMI (body mass index, kg/m^2^) was used. It is based on weight and height and it correlates well with the body’s adipose tissue content, making it possible to assess the prevalence of nutrition disorders (undernourishment or excessive body weight). By correlating the BMI with the risk of all-cause mortality, threshold values were established for adults to define reference ranges for the lowest risk of death. Assessment of the nutritional status of senior citizens was additionally based on Lipschitz’s criteria [31]. Table 3 lists the criteria for assessing the nutritional status based on the Body Mass Index.

The pattern of adipose tissue distribution in the body was assessed on the basis of the waist and hip circumference data and indices based on these data. The WHR (waist to hip ratio: ratio of waist to hip circumference in cm) and WHtR (waist to height ratio: ratio of waist circumference to body height in cm) indices were used. Abdominal obesity is diagnosed when the WHR is equal to or greater than 0.85 in females and equal to or greater than 0.9 in males [22]. A WHtR ≥ 0.5 indicates abdominal obesity irrespective of gender [32]. The type of obesity can also be assessed directly by measuring waist circumference. According to the American National Cholesterol Education Program, abdominal obesity is diagnosed when the waist circumference exceeds 88 cm in women and 102 cm in men. The International Diabetes Federation recommends diagnosing obesity in the European population for waist circumferences exceeding 80 cm in women and 94 cm in men [33,34].

#### 2.2.11. Assessment of Risk of Sarcopenia

For years, sarcopenia was defined as an age-associated syndrome characterised by a reduction in muscle mass and function (muscle strength and physical fitness) [35]. In 2018, the 2nd European Working Group on Sarcopenia (EWGSOP2) [36] modified its 2010 definition by stating that sarcopenia is a muscle disease whose onset may be earlier than in old age. The first stage in diagnostic screening for sarcopenia is the SARC-F questionnaire assessment [19]. This tool comprises 5 questions to assess the following areas: strength, need for assistance while walking, rising from a chair, climbing stairs, and falls. There are three possible answers to each question, awarded 0 to 2 points. The maximum score is 10 points. A score of ≥4 indicates the presence of sarcopenia [36,37]. The present study commenced in 2017. The SARC-F questionnaire was administered to respondents aged 65 years and more in keeping with the then definition of sarcopenia.

#### 2.2.12. Pilot Study

The main study was preceded by a pilot study whose chief aim was to assess the validity of the questionnaires employed, verify correctness of the results obtained and identify potential sources of error. The pilot study enrolled 20 individuals aged 19–64 years and 20 individuals aged 65 years and over. The sample deliberately involved respondents representing different levels of education. Each respondent was tested twice with a minimum of 3 days passing between the interviews. Physical activity was monitored between the first and second visit. Enrolment procedures for the pilot study commenced two weeks before the beginning of the core stage of the pilot study and were based on personal invitations by e-mail or telephone for individuals in the target group. Those who expressed consent to participate and have their personal data processed were enrolled. The interviews were conducted by trained interviewers who paid particular attention to aspects indicated by Oksenberg et al. [38]: the respondent answering a question before the interviewer has finished reading it; requests to repeat or explain a question; giving irrelevant answers not related to the topic; giving relevant and to-the-point answers; giving vague answers such as “it’s hard to tell”; refusing to answer a question.

The interviewers noted any signs of fatigue or impatience on the part of the respondents or other behaviours that could affect data collection quality. In addition, the control team reviewed the collected data in search of differences. If differences were found, a verification procedure was initiated. All diets recorded by the interviewers were verified by qualified dietitians. In case of errors, the interview was returned to the interviewer for verification. If errors occurred regularly for a given interviewer, he or she was directed to take additional training, or was removed from the survey.

Both interviews with a particular respondent were conducted by the same interviewer. The duration of the pilot study was 2 weeks. After the pilot study had been completed and necessary corrections introduced to the questionnaires, the field study commenced.

#### 2.2.13. Methodological Adaptations during the COVID-19 Pandemic

During the COVID-19 pandenic, some methods were modified in accordance with hygeine protocols. A CATI (Computer Assisted Telephone Interview) technique was used to collect the data. All of the questionnaire studies were performed, including 2 studies on food consumption within the last 24 h using the DIETA 5.0 program, with an interval of at least 5 days. Anthropometric data (height, weight and waist circumference) were obtained on the basis of self-measurements by the respondents based on detailed instructions received during the first interview. The data on hip circumference, which is more difficult to self-measure, were dropped. Respondents who had a blood pressure monitor at home reported their own blood pressure. The use of an accelerometer was abandoned.

#### 2.2.14. Field Study

The field study was conducted by a company, selected in a tender procedure, which has experience in conducting CAPI and CATI interviews and could field a team of interviewers across the country. The research company’s task was to carry out the study of a sample provided by the Medical University of Warsaw. The Interviewers’ tasks involved enrolling the selected respondents, obtaining all necessary consent to conduct the study, and visiting the respondents at their places of residence twice in order to conduct the survey and tests scheduled for a given visit (during the COVID-19 pandemic, this was replaced by conducting two telephone interviews). All study data were regularly submitted to the study staff at the Medical University of Warsaw. Before the field visit stage, all interviewers were equipped with identical sets of instruments by the Medical University of Warsaw to ensure standardised measurements. The sets consisted of a scale, a medical measuring tape, a set square and a sphygmomanometer. The interviewers also completed detailed training at the Medical University of Warsaw (MUW) covering all planned methods and measurements using the dedicated equipment, and received instructional materials with detailed guidance prepared by the study staff. The work of interviewers was supervised by a field coordinator representing the research company; his responsibilities also included passing on all information related to the implementation of the study to the project coordinator at the MUW. Throughout the study, interviewers could rely on technical support from the MUW study staff. In return for their participation, each respondent received a detailed analysis of their daily diet compiled individually on the basis of the survey responses and measurements. Each respondent also received an individually directed brochure on the principles of rational nutrition and a website address where (s)he could find information about the level of physical activity and dietary mistakes made, prepared on the basis of individual research results. In addition to contributing to health benefits and the prevention of diet-related disease, this activity was intended to encourage respondents to participate in the study. All respondents who provided all of the information requested of them received a voucher worth 50 PLN. 

#### 2.2.15. Quality Control

The study data were processed by two independent quality control systems. Basic (standard) quality control measures were implemented by the research company according to an approved scheme. The following information was verified: whether an interview actually took place, what answers were provided in response to key questions, and what materials were supplied by the interviewer. The MUW team carried out an independent quality control procedure, to verify that each questionnaire was completed correctly.

Data integrity was also verified. Analyses of the data submitted were conducted continuously, making it possible to monitor the progress of the field study. Outliers were subject to further verification and reported for more extensive quality control.

#### 2.2.16. Qualitative Study

Individual (Individual In-depth Interview, IDI) and group-based (Focus Group Interview, FGI) qualitative surveys were carried out in order to assess respondents’ knowledge about nutrition and sources of information and optimal channels for disseminating nutrition-related information.

#### 2.2.17. Individual In-Depth Interviews

The underlying principle in individual in-depth interviews (IDI) is that the interviewer talks to one person at a time who can speak freely on topics provided by the interviewer. The aim is to obtain detailed opinions and information on a topic or explain a phenomenon. During an interview, the respondent can formulate their responses freely, and the interviewer can ask additional questions about details at any moment. A total of 80 individual interviews were carried out for the study (40 individuals in each age group, i.e., 19–64 years (NHP-A) and 65+ years (NPH-S) at 10 diverse localities around the country (4 rural and 6 urban areas), with 8 interviews carried out at each locality (4 in NHP-A and 4 in NHP-S, including two interviews with male participants and two interviews with female participants in each group). The interviews were recorded and subsequently transcribed by the interviewers.

#### 2.2.18. Focus Group Interviews

Focus group interviews (FGI) are qualitative interviews of groups of respondents. This technique assumes interactions between the respondents as an additional source of information. The protocol of a focus group interview is a list of issues and questions that the moderator should discuss during the meeting. A protocol contains certain suggestions and instructions rather than an exhaustive list of issues and questions. During a focus group meeting, the moderator may adjust the content to the course of the discussion in order to obtain more in-depth responses. Focus group interviews were carried out in 10 groups (5 each among adults and senior citizens) of 6 to 8 individuals. Within each age group, two interviews were carried out in rural areas and 3 in urban settings. Respondents were selected to participate in focus group interviews on the basis of their availability. Because of the COVID-19 pandemic, 50 individual and 10 group interviews were carried out online. During the pandemic, both individual and group interviews were carried out as videoconferences over the Internet. This change had an impact on the availability of respondents, especially those aged 65 years and older. Only respondents with an Internet connection and appropriate online communication skills could take part.

## 3. Data Acquisition and Processing

During the study, quantitative data were acquired in a continuous manner in two independent systems developed for the purposes of the project. One system was technical and served to enable on-going verification of study data and formulation of nutrition-related recommendations for the respondents. Data were recorded in SQL databases and processed in R ver. 3.6.3 (R Core Team., Vienna, Austria). The other system served analytical purposes and used IBM SPSS ver. 25 & 26 (SPSS Inc., Chicago, IL, USA). Data for the technical and analytical systems were primarily processed in dedicated applications for analysing respondents’ daily diets (Dieta 5.0 and 6.0, National Food and Nutrition Institute, Warsaw, Poland) and physical activity (ActiLife Version. 6.11.9; Actigraph), and subsequently exported and fed into R and SPSS. 

Errors were identified in an on-going manner and the research company was immediately notified to enable correction. The corrected data were re-entered and subjected to further quality control procedures. All data related to dietary evaluation were verified manually by a team of dietary experts. 

## 4. Changes to Study Methodology during the COVID-19 Pandemic—Summary

The total number of respondents who completed the study before the onset of the COVID-19 pandemic in Poland (4 March 2020) was 3217 out of 4000 as had been planned (1,684 individuals from NHP-A and 1533 individuals from NHP-S).

The introduction of public health restraints made further direct examination and face-to-face interviewing of the respondents impossible, and the study team were forced to modify study methods and procedures. It was decided that further quantitative surveys would be conducted exclusively by telephone, while qualitative investigations—individual and group interviews—took place online using videoconferencing systems.

The study was resumed in June 2020. The initial stage was interviewer training, which was conducted on-line. 

Due to the pandemic, not all procedures could be performed by interviewers as planned. These were, namely, measurements of anthropometric indices, blood pressure and physical activity levels. According to the study’s original methodology, these examinations would be performed by interviewers with the aim of obtaining as standardized data as possible. The assessment of actual physical activity using an accelerometer was also an important aspect. 

During the pandemic, respondents were interviewed on the phone. All survey components were carried out, including two 24-h dietary recall surveys supported with Dieta 5.0 software at least 5 days apart. The anthropometric parameters, recorded as self-reported by respondents, were limited to body weight, height and waist circumference, which respondents reported after having carried out the measurements themselves. Hip circumference was not required as this parameter is more difficult for respondents to measure on their own. Respondents who had a sphygmomanometer at home reported the blood pressure readings they had obtained on their own. Accelerometers were not used. 

## 5. Discussion

This is the first representative study of nutrition and nutritional status of the adult Polish population to have used such a wide array of research techniques. While the first representative nutrition study in Poland from 2000 did use 24-hdietary recall, the interviews were only carried out once for each respondent [39]. Thus, the results could not be used for assessing exposure to substances hazardous to health. In other studies conducted in Poland (WOBASZ I and WOBASZ II) only one day 24-h dietary recall was used. And, moreover, have focused mostly on cardiovascular disease risk factors [40]. Repeated 24-h dietary recall interviews have been used in a number of cross-sectional studies of nutrition in the world; as a result, the data from Poland can be compared to data from other countries. This procedure has been employed in studies from Belgium, Austria, Germany, the USA and other countries [41,42]. In our study, the survey of nutrition using 24-h dietary recall [23,24] was supported with the Food Frequency Questionnaire (FFQ) [18], which furnishes data on food consumed occasionally.

Data collected during the present study also included the composition and dosages of dietary supplements used by the respondents, which makes it possible to calculate the contribution of dietary supplements to the consumption of vitamins and minerals. A notable component of the study is the first representative assessment of physical activity levels, with data gathered not only through interviews but also via accelerometers, which have been used in other studies and so will allow for comparisons [38]. The study data will also provide representative information about overall health of the study population, the prevalence of food allergies and the risk of sarcopenia in senior citizens, as well as respondents’ awareness of food and nutrition, which will help adjust nutrition education to the needs of the adult Polish population.

## 6. Strengths and Limitations of the Study

Unlike previous studies on nutrition in the Polish population [3,40,43], our study takes into account the methodology of nutrition research under EFSA guidelines [13,15] and Polish Academy of Sciences guidelines, [15,16,17,18] and the methodology of studies from other countries [41,42], to a much greater extent. This is the first Polish study to analyse such a wide range of aspects using a combination of quantitative and qualitative methods and a variety of research techniques to assess nutrition patterns, physical activity, nutritional status and overall health. It is the first representative study in Poland to use double interviews on food consumption in the previous 24 h, as well as recording participants’ physical activity with accelerometers and the risk of sarcopenia in the older population. Another strength of the present study is its use of professional software to record dietary information, guaranteeing high measurement quality and an opportunity to analyse nutrients composition of the diet. The software used [24] is the only tool recommended in Poland for conducting scientific research in the field of dietary assessment. It is also worth noting that the anthropometric data taken before the COVID-19 pandemic were actual measurements made by trained interviewers and are not declared data.

However, some limitations are of note. First, the duration of the first interview was quite long. Due to the number of tools used, the first study visit was completed in approximately 90 min. Such a long interview time requires the respondent to maintain interest and concentration in providing answers. The pilot study made it possible to determine an optimal duration of the interview which could achieve the study’s goals in a length of time acceptable for the respondent. Before starting the survey, the interviewers practiced the procedure to be able to conduct interviews within the determined timeframe.

Those methods in the study which are based on the respondent’s memory may be a source of errors such as the possibility that respondents may forget the details of the food they have eaten. In the case of our study, conducting interviews at the respondent’s home made it possible to partially eliminate this problem by reducing stress—the respondents were at their own home—and the opportunity for the interviewer to achieve more precise estimation of portion sizes.

An unexpected, unforeseeable limitation of the study was the COVID-19 pandemic and its hygiene restrictions. Suspension of the research until restrictions were lifted was not possible due to the strictly defined duration of the study. In order to complete the project, it was necessary to introduce changes to the research methodology. As a result, anthropometric data for some of the respondents is based on self-reports. It was impossible to perform an accelerometer test, and no data on hip circumference were collected.

## 7. Conclusions

The methods used in the study allowed for obtaining unique data on nutrition and nutritional status, physical activity level and the prevalence of non-infectious chronic diseases with particular regard to obesity and allergy, and to estimate the prevalence of suspected sarcopenia among respondents aged 65 years or more. 

The study findings will be used to design appropriate needs-based education and for research purposes. Data from the repeated 24-h dietary recall interview supported with the food frequency questionnaire will make it possible to assess exposure to undesirable substances in the diet. Key findings will be prepared for publication in 2021. They will initially include an evaluation of nutrition, assessment of nutritional status with identification of risk factors for nutritional status disorders, assessment of the level of physical activity and assessment of the level of nutrition awareness. The results will be presented separately for data collected before and during the COVID-19 pandemic.

Due to its duration, from 2017 to 2020, the present study is uniquely valuable. Impacts of the COVID-19 pandemic on the nutritional behaviour of the studied population will be published soon. The simultaneous application of a number of research methods gives a broad overview of the situation in the field of nutrition, nutritional status and the level of physical activity—factors which determine a society’s health. Conclusions from these studies may be important for building a public health strategy that reduces the risk of lifestyle diseases, mainly obesity, type 2 diabetes, cardiovascular diseases, and some cancers.

## Figures and Tables

**Table 1:** Quantitative methods /study procedures employed in the study.

Method/Procedure	Description/Comments	Source
Visit I, approximate duration 90 min		
1. Respondents’ overall health questionnaire	13 questions about the presence of chronic medical conditions	Author’s questions
2. Arterial blood pressure measurement	After 5 min’ rest (see description below)	According to universally adopted methodology [20,21]
3. Anthropometric measurements	Body weight, Height, Circumference: waist, hips	According to WHO guidelines [22]
4. Survey of beliefs on nutrition and food	19 statements about the role of food components and food processing methods	Based on the KomPan questionnaire [18]
5. 24-h dietary recall interview	Dieta 6, a type of software used in nutrition-related research in Poland	National Food and Nutrition Institute in Warsaw [23,24]
6. Survey of consumption of dietary supplements	5 questions about consumption of dietary supplements (name, composition, time of consumption, reason for consumption)	Author’s questions
7. Survey of eating habits	7 questions, with topics including the number of meals in a day, consumption of vegetables, fruit and fast food on a typical day, any diets	Based on the KomPan questionnaire [18]
8. Survey of food consumption frequency	25 questions about the frequency of consumption of various food products/product groups	Based on the KomPan questionnaire [18]
9. Survey of physical activity	9 questions	IPAQ short version questionnaire (last 7 days) [25] 2 questions from the KomPan questionnaire [18]
10. Survey on selected lifestyle elements and self-assessment of nutritional knowledge and nutrition	8 questions	Based on the KomPan questionnaire [18]
11. Survey of food allergies	16 questions about symptoms, symptom frequency, sensitising foods, and who established the diagnosis of food allergy and on what basis	Author’s questions
12. Survey of sleep quality	8 questions about the duration of sleep, awakening at night, overall well-being after a bad night	Athens Insomnia Scale [26]
13. Survey of socio-demographic data	6 questions: place of residence, income, level of education, employment	Based on the KomPan questionnaire [18] + original questions
14. Assessment of risk of sarcopenia	5 questions	Simple Questionnaire to Rapidly Diagnose Sarcopenia (SARC-F) [19]
15. Priming and handing over devices for measuring physical activity	Instructions on using the device for measuring physical activity	Author’s questions
16. Measurement and assessment of physical activity levels	Actigraph GT3X accelerometer	Software: ActiLife Version. 6.11.9; Actigraph
Visit II, approximate duration 45 min		
17. 24-h dietary recall interview	Dieta 6, a type of software used in nutrition-related research in Poland	National Food and Nutrition Institute in Warsaw [23,24]
18. Arterial blood pressure measurement	After 5 min’ rest (see description below)	According to universally adopted methodology [20,21]
19. Recovery of devices for measuring physical activity		

**Table 2:** Scheme for anthropometric measurements depending on respondents’ overall health and physiological status.

Variable	Respondent is Wheelchair-Bound/Bed-Ridden	Respondent Cannot Stand Up	Respondent’s Body Weight Exceeds the Upper Body Weight Limit of 120 kg	The Woman Is More Than 20 Weeks Pregnant
Body weight	self-reported	self-reported	self-reported	self-reported
Height	self-reported	self-reported	measured	measured
Waist circumference	self-reported	self-reported	measured	self-reported pre-pregnancy values
Hip circumference	self-reported	self-reported	measured	self-reported pre-pregnancy values

**Table 3:** Criteria for assessing nutritional status based on the Body Mass Index.

Interpretation of BMI Values (kg/m^2^) in the Adult Population (WHO) [30]		Interpretation of BMI Values (kg/m^2^) in the Senior Citizen Population (Lipschitz Criteria) [31]	
Underweight	<18.49	Underweight	<22
Normal weight	18.5–24.9	Normal weight	22–27
Overweight	25.0–29.9	Overweight	>27
Obesity	≥30	-	-

## Supplementary Materials

The following are available online at https://www.mdpi.com/article/10.3390/nu13082568/s1, Figure S1: CAPI sampling methodology, Figure S2: CATI sampling methodology.
