# Supplementary figures and images for: A National Study of Nutrition and Nutritional Status of the Adult Polish Population in the Years 2017–2020 before and during the COVID-19 Pandemic—Design and Methods
Source: Nutrients. 2021 Jul 27;13(8):2568. doi: 10.3390/nu13082568 (PMC8398539; doi:10.3390/nu13082568)

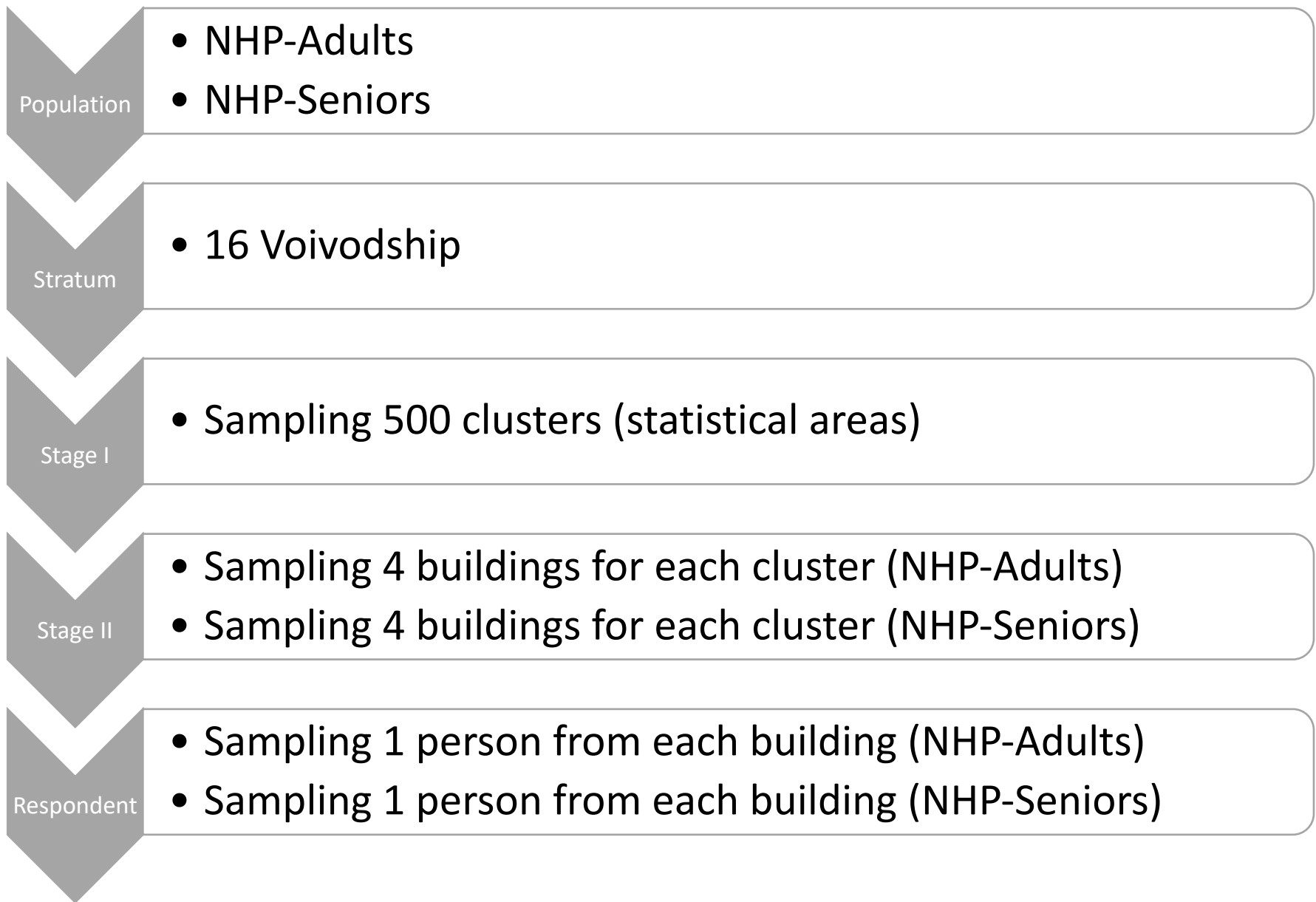

Figure S1: CAPI sampling methodology

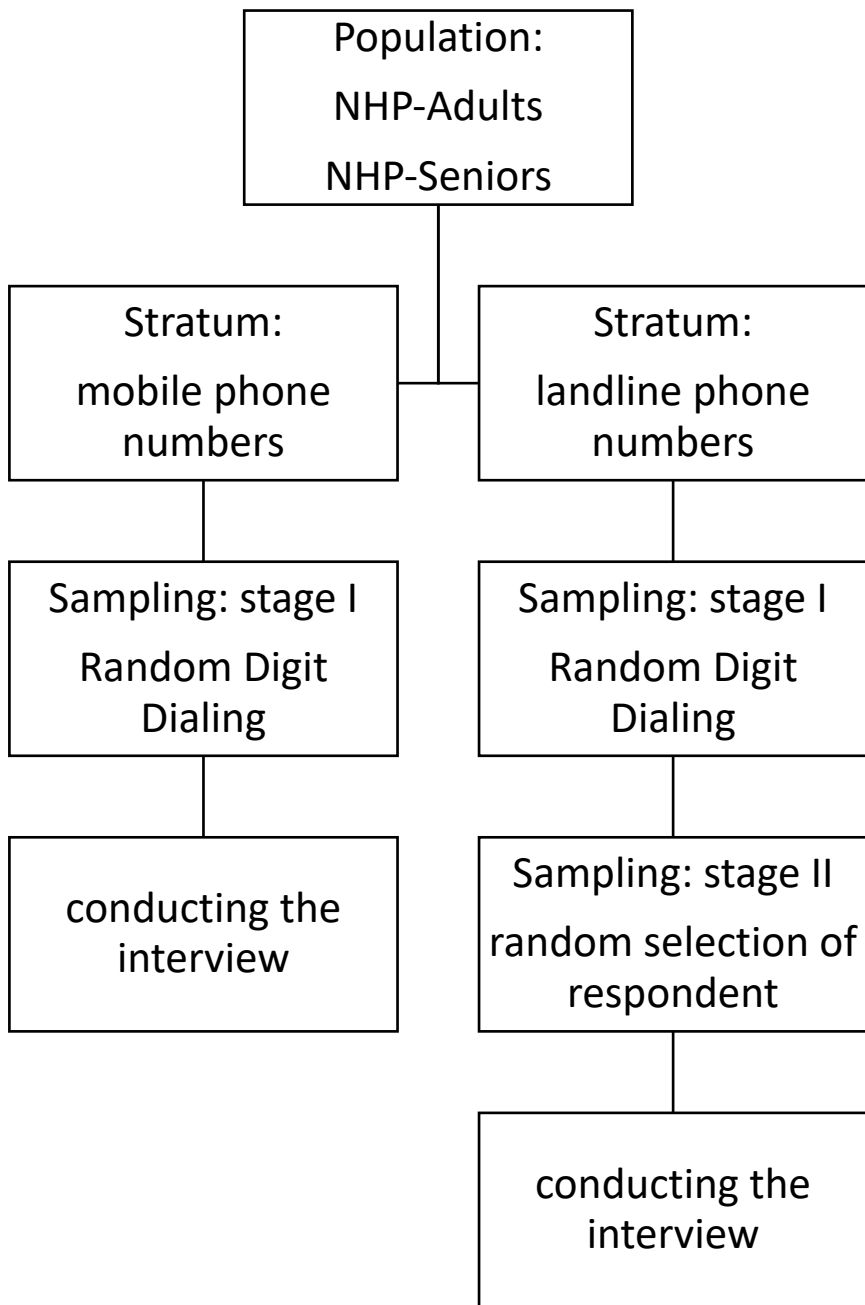

Figure S2: CATI sampling methodology

Supplement: Supplementary file 1 [file nutrients-13-02568-s001.zip › nutrients-1271903-supplementary.pdf]
